# Biportal endoscopic bilateral decompression in lumbar spinal stenosis: a 3-year retrospective cohort study

**DOI:** 10.3389/fsurg.2025.1601944

**Published:** 2025-05-30

**Authors:** Dongyue Li, Yunzhong Cheng, Peng Yin, Qingjun Su

**Affiliations:** Orthopaedic Department, Chaoyang Hospital Affiliated to Capital Medical University, Beijing, China

**Keywords:** biportal endoscopy, bilateral decompression, lumbar spinal stenosis, clinical efficacy, radiographic findings

## Abstract

**Background:**

Biportal endoscopic bilateral decompression (BEBD) has gained recognition for treating lumbar spinal stenosis (LSS) through preservation of posterior spinal structures while achieving bilateral neural decompression. However, the relationship between postoperative radiographic findings and clinical outcomes remains unclear. This study investigates clinical efficacy, radiographic findings, and their potential correlations following BEBD.

**Methods:**

A retrospective cohort analysis of 51 LSS patients undergoing BEBD (January 2020–December 2021) was conducted. Intraoperative parameters, complications, and clinical outcomes [Visual Analog Scale (VAS), Oswestry Disability Index (ODI), Modified Macnab criteria] were evaluated preoperatively, at 1 month, and final follow-up. Radiographic parameters included medial facetectomy surface angle (MFSA), facet joint preservation rate (FJPR), lateral recess decompression rate (LRDR), dural sac cross-sectional area expansion rate (DSCAER), and segmental range of motion (SROM).

**Results:**

The procedure demonstrated the mean operative time of 93.6 ± 13.7 min, with follow-up 36–60 months (mean 42.5 ± 6.7 months). Clinically, lower back pain (VAS: 5.9 ± 0.9–2.3 ± 0.6 at 1 month; 0.6 ± 0.5 final) and leg pain (6.8 ± 0.9–1.7 ± 0.6 at 1 month; 0.5 ± 0.6 final) showed sustained, statistically significant reductions (*P* < 0.05). Functional recovery was marked by ODI improvements from 64.5 ± 7.5 preoperatively to 26.1 ± 2.8 (1 month) and 11.0 ± 2.3 (final) (*P* < 0.05), with 88.24% (45/51) achieving excellent/good outcomes by modified Macnab criteria. Radiographically, MFSA remained <90°, FJPR exceeded 70%, and DSCAER expanded by 95.19 ± 22.5% (*P* < 0.05), while SROM stability was preserved (*P* > 0.05). Notably, no radiographic findings correlated with clinical outcomes stratification (*P* > 0.05), underscoring the multifactorial nature of postoperative success.

**Conclusions:**

BEBD demonstrates significant clinical improvement in LSS patients, with marked DSCA expansion and preserved FJ stability. The technique achieves effective bilateral decompression with preserved biomechanical stability. Radiographic findings showed no correlation with clinical success, indicating multifactorial postoperative influences.

## Introduction

Surgical intervention is typically warranted when lumbar spinal stenosis (LSS) significantly impairs patients' activities of daily living (1). Traditional open decompression strategies, notably posterior lumbar interbody fusion (PLIF) and transforaminal lumbar interbody fusion (TLIF), remain the cornerstone of surgical management, achieving therapeutic efficacy through radical resection of compressive structures and segmental stabilization. However, mounting evidence highlights the inherent trade-offs of these techniques: extensive disruption of posterior tension bands (supraspinous/interspinous ligaments, laminae) predisposes to iatrogenic instability; aggressive paraspinal muscle retraction correlates with postoperative atrophy and chronic myofascial dysfunction; and the biomechanical consequences of rigid fixation accelerate adjacent segment disease (2, 3).

The paradigm shift toward minimally invasive spine surgery has catalyzed the refinement of endoscopic techniques, with biportal endoscopic bilateral decompression (BEBD) emerging as a disruptive innovation for LSS management (4–9). This technique synergizes the advantages of unilateral laminotomy with bilateral visualization, enabling circumferential decompression under saline-mediated magnification while preserving dynamic stabilizers. Contemporary series report comparable pain relief to open techniques with superior preservation of paraspinal musculature (10). Nevertheless, critical knowledge gaps persist regarding the predictive value of quantitative imaging biomarkers—including facet joint (FJ) preservation, lateral recess (LR) decompression, and dural sac cross-sectional area (DSCA) —for stratifying surgical candidates and prognosticating functional recovery.

While preliminary clinical studies demonstrate satisfactory outcomes with this technique, critical knowledge gaps persist regarding radiological correlates of surgical success. This retrospective cohort study addresses some critical questions in single-level LSS management: clinical prognostic profiles, imaging parameters, and their interrelationships. By analyzing longitudinal associations between multidimensional radiographic findings and validated clinical outcomes, this investigation seeks to establish evidence-based benchmarks for optimal patient selection and outcome stratification in BEBD procedures.

## Materials and methods

### Clinical data

A retrospective cohort analysis was conducted on patients with LSS treated with BEBD in our department from January 2020 to December 2021. Patients meeting the following criteria were included in this study. **Inclusion criteria**: (1) Presenting with nerve root symptoms in bilateral lower extremities or neurogenic intermittent claudication; (20 Not responsive to conservative treatment for 3–6 months and above; (3) Diagnosed with LSS by preoperative imaging examinations, and the radiological findings consistent with clinical manifestations; (4) The clinical symptoms were largely attributed to a single segment; (5) No instability of the lumbar spine in the culprit segment (the angular difference between the lower and upper endplates in the affected segment <10° or the migration distance of the vertebral body <4 mm on the preoperative x-ray image in hyperextension and hyperflexion) (11). **Exclusion criteria**: (1) Multi-segment LSS; (2) Combined with lumbar spondylolisthesis, lumbar spine instability, or degenerative scoliosis; (3) History of lumbar surgery; (4) Lumbar tuberculosis, tumors, intervertebral disc infection, ankylosing spondylitis, and fractures. The surgeon has performed over 100 cases of this procedure, demonstrating extensive experience and technical mastery.

### Surgical procedures

All patients received BEBD at a single segment. Choice of the left or right side for biportal endoscopy: (I) The left side was chosen for patients with bilateral symptoms of the same severity; (II) The more severely affected side was chosen for bilateral symptoms of unequal severity. The patients took a prone position after general anesthesia. The responsible level was located using the C-arm system. A longitudinal incision was made at 1.5–2.0 cm from the midline line, at 1.5 cm above and below the responsible level, respectively. The proximal incision was intended for the observation port, which ran for a length of about 0.4 cm, and the 0° spinal endoscope was inserted. The distal incision running for a length of about 1.0 cm was intended for operation port, through which the operating equipment was inserted and delivered to the root of the spinous process of the superior vertebra by passing through the paravertebral muscle. The perfusion system was turned on, with the fluid level maintained at 50 cm above the incision plane. A plasma radio-frequency electrotome was inserted via the operation port to dissociate the soft tissues from the vertebral plate and the ligamentum flavum. Portions of bone substance were removed from the laminar margin and the medial aspect of the inferior articular process to expose the upper and lower borders of the ligamentum flavum. The ligamentum flavum was dissociated and resected to expose the dural sac. Part of the medial border of the superior articular process was resected laterally to expose the nerve root. The orientations of the endoscope and the operation port were adjusted to perform contralateral intraspinal decompression. Part of the bone substance in the spinous base was resected to create enough space for contralateral undermining decompression. Contralateral hypertrophied ligamentum flavum was resected using the same method as in ipsilateral decompression. Part of the medial facet joint was resected, if necessary, to expose the contralateral nerve root. Be cautious to protect the facet joint and avoid over-resection that might otherwise threaten the spinal stability. Exploration was performed to ensure that the bilateral nerve roots were fully relaxed without compression and that the dural sac was normally undulated. The operating equipment and the endoscope were withdrawn after confirming the success of hemostasis. The residual rinse solution was drained by squeezing with both hands. A drainage tube was indwelled at the operation port. All surgeries were performed by one surgeon. The patients stayed in bed on the day of surgery, and the drainage tube was removed the next day. The patients were encouraged to ambulate with waist support and avoid heavy physical labor or exercise within 3 months after surgery.

### Clinical outcome measures

Perioperative parameters encompassing demographic characteristics (age, gender), surgical details (operated spinal segment, operative duration, intraoperative blood loss), and postoperative complications were systematically documented. Pain intensity in the lower back and lower extremities was quantitatively evaluated using the visual analog scale (VAS), while functional recovery and quality of life were assessed through the Oswestry Disability Index (ODI). These evaluations were performed at three distinct time points: preoperatively, 3-month postoperatively, and during the final follow-up examination. Clinical outcomes were ultimately classified according to the modified MacNab criteria at the last follow-up interval.

### Radiological evaluation

**Figure 1:**
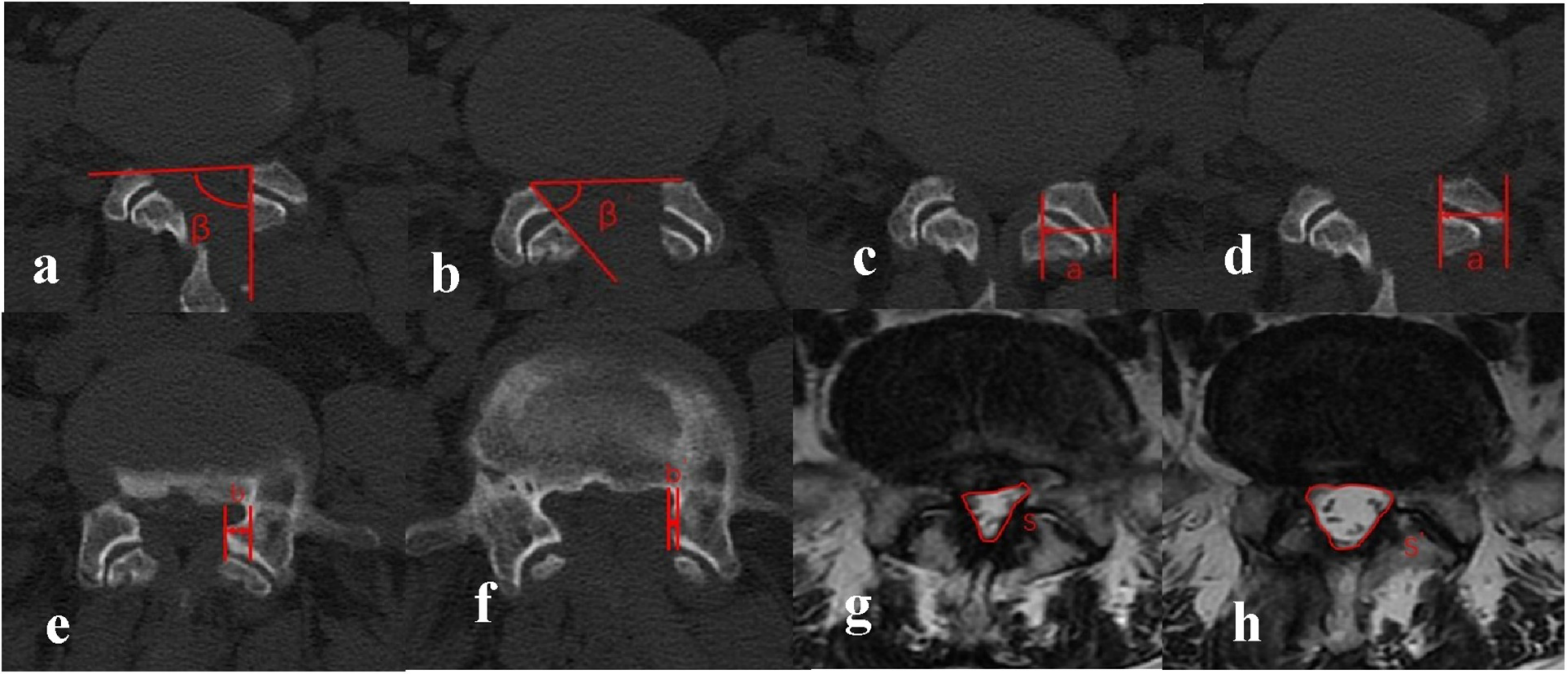
**(a,b)** medial facetectomy surface angle (MFSA): included angle between the line connecting the ventral vertices of the facet joints (FJ) on the two sides and the surgical tangent line of FJ, which was defined as the angle of MFS. β indicated angle at the ipsilateral side and β’ indicated angle at the contralateral side. **(c,d)** Facet Joint Preservation Rate (FJPR): The preoperative FJ width was denoted by a, and the postoperative FJ width was denoted by a’. The preservation rate of FJ = a’/a × 100%. **(e,f)** Lateral Recess Decompression Rate (LRDR): The preoperative distance from the medial border of the superior FJ to the medial border of the pedicle of vertebral arch was denoted by b, and the postoperative distance was denoted by b’. The decompression rate of LR = b’/b × 100%. **(g,h)** Dural Sac Cross-Sectional Area Expansion Rate (DSCAER): DSCAmeasured on the lumbar spine MRI was denoted by S, and the postoperative area was denoted by S’. The expansion rate of DSCA = S’/S × 100%.

All patients underwent comprehensive imaging assessments comprising dynamic lumbar x-rays (hyperextension and hyperflexion) and three-dimensional computed tomography (3D-CT) immediately postoperatively, followed by lumbar spine magnetic resonance imaging (MRI) reexamination at the 3-month follow-up. Quantitative analysis of imaging parameters was performed using standardized techniques by a collaborative team of one orthopedic surgeon and one radiologist, with final values derived from averaged measurements. Key metrics included (Figure 1):

(1) Medial Facetectomy Surface Angle (MFSA), defined as the acute angle between the medial resection plane of the residual inferior facet joint and the tangent line connecting bilateral superior facet joints on axial CT;
(2) Facet Joint Preservation Rate (FJPR), calculated as (postoperative FJ width/preoperative width) × 100% on axial CT;
(3) Lateral Recess Decompression Rate (LRDR), expressed as [(preoperative distance from the medial superior facet joint border to the pedicle – postoperative distance)/preoperative distance] × 100% on axial CT;
(4) Dural Sac Cross-Sectional Area Expansion Rate (DSCAER), quantified by [(postoperative DSCA – preoperative DSCA)/preoperative DSCA] × 100% using axial MRI measurements of the dural sac boundaries; and
(5) Segmental range of motion (SROM), determined by angular differences between hyperextended and hyperflexed positions on lateral lumbar radiographs.

This protocol ensured systematic evaluation of anatomical restoration, neural decompression efficacy, and dynamic stability through multimodal imaging correlation.

### Statistical analysis

All analyses were conducted using SPSS 19.0. Continuous variables are presented as mean ± standard deviation (SD). Between-group comparisons were performed using independent-samples *t*-tests, while within-group preoperative and postoperative differences were assessed via paired-samples *t*-tests. For longitudinal evaluation of parameters measured at multiple timepoints, one-way repeated-measures analysis of variance (ANOVA) was applied. Bivariate correlations between imaging metrics and clinical outcomes were quantified using Pearson's correlation coefficients. A two-tailed *p*-value <0.05 defined statistical significance.

## Results

### Clinical outcomes

According to the inclusion and exclusion crite­ria, a total of 51 patients with LSS were enrolled in this study. All patients (22 males, 29 females; mean age 63.35 ± 7.76 years, range 50–78) successfully underwent single-level BEBD, with procedures performed at L3/4 (*n* = 4), L4/5 (*n* = 29), and L5/S1 (*n* = 18). Mean operative time was 93.59 ± 13.73 min (range 75–125 min) with intraoperative blood loss averaging 31.30 ± 8.59 ml (range 20–50 ml). Three perioperative complications were documented: one intraoperative dural tear managed with primary repair and postoperative conservative measures (intravenous hydration, Trendelenburg positioning), one case of transient lower limb paresthesia resolved through pharmacologic intervention, and one symptomatic epidural hematoma treated non-operatively with low-dose corticosteroids and mannitol therapy. Notably, no instances of neurovascular injury, surgical site infections, or myelopathic sequelae were observed. All incisions healed primarily without delayed complications.

### Functional outcomes

All patients completed a minimum 3-year follow-up period, with postoperative surveillance spanning 36–60 months (mean duration: 42.5 ± 6.7 months). As summarized in Table 1, preoperative, 3-month postoperative, and final follow-up assessments of VAS scores for lower back/leg pain and ODI were analyzed. Compared to preoperative baselines, both VAS scores and ODI demonstrated statistically significant reductions at 3 months post-surgery (*P* < 0.05). Furthermore, these metrics exhibited sustained progressive declines over time, reaching significantly lower values at the final follow-up compared to the 3-month postoperative assessment (*P* < 0.05). At the last follow-up, functional outcomes evaluated using the modified MacNab criteria revealed excellent results in 35 patients (68.63%), good outcomes in 10 (19.61%), fair outcomes in 6 (11.76%), and no poor outcomes, yielding an overall excellent-to-good rate of 88.24% (45/51) (Table 1).

**Table 1:** Data of patients, VAS, ODI, and modified MacNab score (mean ± SD).

Index	*n* = 51	*F* *	*P* *
Age (year)	63.35 ± 7.76		
Surgical level			
L3/4	4		
L4/5	29		
L5/S1	18		
Operation time (min)	93.59 ± 13.73		
Estimated blood loss (ml)	31.30 ± 8.59		
Follow up (month)	42.5 ± 6.7		
VAS score for back pain			
Preoperative	5.87 ± 0.94	396.683	0.000
3 month after surgery	2.30 ± 0.66		
Last follow up	0.61 ± 0.54		
VAS score for leg pain			
Preoperative	6.78 ± 0.89	902.184	0.000
3 month after surgery	1.67 ± 0.60		
Last follow up	0.50 ± 0.59		
ODI score			
Preoperative	64.48 ± 7.47	1,465.867	0.000
3 month after surgery	26.09 ± 2.80		
Last follow up	10.96 ± 2.30		
Modified MacNab score			
Excellent	35		
Good	10		
Fair	6		
Poor	0		

* The same data is measured multiple times, and one-way repeated measures ANOVA is applied.

### Radiological findings

**Figure 2:**
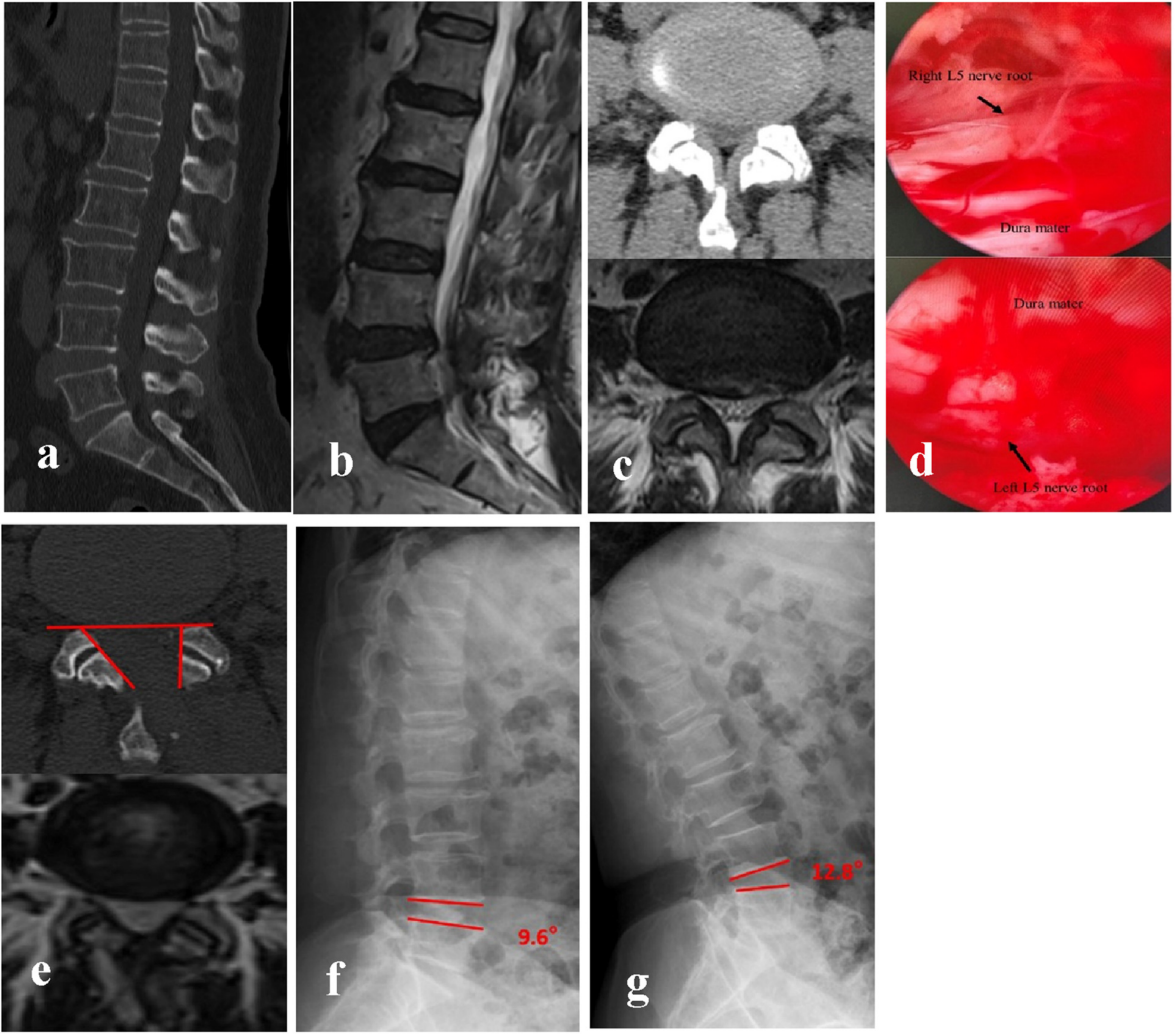
BEBD was performed via the left-sided approach for bilateral decompression at the L4/5 segment. **(a–c)** Preoperative CT and MRI demonstrated bilateral lateral recess stenosis at L4/5, resulting in significant compression of bilateral L5 nerve roots. **(d)** Intraoperative visualization confirmed adequate decompression and relaxation of bilateral L5 nerve roots following the procedure. **(e)** Postoperative CT revealed partial resection of the left medial FJ (FJPR: 73.4%) and satisfactory preservation of the right FJ (FJPR: 98.1%). Bilateral LR were sufficiently decompressed. MFSA measured 88.9° (ipsilateral) and 54.6° (contralateral). Postoperative MRI at 3 months after surgery documented a 98% increase in DSCAER, confirming sustained decompression. **(f,g)** Dynamic flexion-extension radiographic evaluation at final follow-up confirmed preserved segmental stability at the L4/5 level, with no evidence of abnormal motion or pathological listhesis observed.

The radiological analysis of 51 patients demonstrated significant postoperative changes in key anatomical parameters following BEBD (Table 2). On the ipsilateral side, MFSA measured 86.51 ± 2.04°, notably greater than the contralateral measurement of 60.33 ± 3.36° (*P* < 0.05). FJPR showed a marked disparity between sides, with 73.13 ± 3.57% on the ipsilateral vs. 93.41 ± 2.91% on the contralateral (*P* < 0.05). Similarly, LRDR revealed significantly more improvement on the ipsilateral side (30.07 ± 2.96%) compared to the contralateral side (8.33 ± 1.48%) (*P* < 0.05). DSCAER exhibited substantial improvement, increasing from 68.91 ± 6.81 mm^2^ preoperatively to 133.13 ± 7.15 mm^2^ postoperatively, representing a 95.19 ± 22.54% expansion (*P* < 0.05). Notably, the operated segment maintained functional integrity with no significant alteration in SROM compared to preoperative measurements (*P* > 0.05), and no instances of postoperative instability were observed during follow-up. A representative case illustrating these morphological changes is presented in Figure 2.

**Table 2:** Radiological parameters of BELD treatment for LSS (mean ± SD).

Parameter	*n* = 51	*t*	*P*
MFSA (°)			
Ipsilateral	86.51 ± 2.04	45.951*	0.000*
Contralateral	60.33 ± 3.36		
FJPR (%)			
Ipsilateral	73.13 ± 3.57	33.401*	0.000*
Contralateral	93.41 ± 2.91		
LRDR (%)			
Ipsilateral	30.07 ± 2.96	45.775*	0.000*
Contralateral	8.33 ± 1.48		
DSCAER (mm^2^)			
Preoperative	68.91 ± 6.81	40.841^*Δ*^	0.000^*Δ*^
3 month after surgery	133.13 ± 7.15		
Increasing (%)	95.19 ± 22.54		
SROM			
Preoperative	6.49 ± 0.57	0.144^*Δ*^	0.886^*Δ*^
Last follow up	6.50 ± 0.41		

MFSA, medial facetectomy surface angle; FJPR, facet joint preservation rate; LRDR, lateral recess decompression rate; DSCAER, dural sac cross-sectional area expansion rate; SROM, segmental range of motion.

* Independent sample *t*-test, ^Δ^Paired sample *t*-test.

### Correlation between clinical outcomes and radiological parameters

To analyze the correlation between clinical outcomes, functional outcomes, and imaging parameters, patients were stratified into two subgroups based on the modified MacNab criteria: the excellent/good outcome group and the fair/poor outcome group. Imaging parameters were compared between these subgroups. Although no significant differences were observed in imaging parameters between the two groups (*P* > 0.05), the excellent/good outcome group showed a higher LRDR on the ipsilateral side (30.83 ± 2.99 vs. 29.80 ± 2.59, *P* = 0.167) and a greater increase in DSCAER (95.98 ± 22.54 vs. 86.80 ± 30.16, *P* = 0.412). However, no significant differences in imaging parameters were found between patients with excellent/good outcomes and those with fair/poor outcomes following BEBD (*P* > 0.05) (Table 3). Pearson's correlation analysis was conducted between imaging parameters—including MSFA, FJPR, LRDR, DSCAER, and SROM—and clinical outcomes, such as VAS scores for lower back and leg pain and ODI. No significant correlations were identified between imaging parameters and clinical outcomes (*r* < 0.3, *P* > 0.05).

**Table 3:** Comparison of radiological parameters between excellent/good outcomes and fair/poor outcomes according to the modified MacNab criteria (mean ± SD).

Item	Excellent/good outcomes (*n* = 45)	Fair/poor outcomes (*n* = 6)	*t*	*P* *
Angle of MFS (°)				
Ipsilateral	86.58 ± 1.97	86.00 ± 2.92	0.597	0.553
Contralateral	60.45 ± 3.51	60.00 ± 2.01	0.279	0.781
Residual rate of FJ (%)				
Ipsilateral	73.85 ± 3.44	75.60 ± 4.39	1.639	0.109
Contralateral	93.55 ± 2.86	93.00 ± 3.54	0.396	0.694
Decompression rate of LR (%)				
Ipsilateral	30.83 ± 2.99	29.80 ± 2.59	1.407	0.167
Contralateral	8.38 ± 1.40	8.10 ± 1.92	0.109	0.914
DSCA (mm^2^)				
Preoperative	68.18 ± 7.04	67.40 ± 9.50	0.642	0.524
3 month after surgery	133.33 ± 6.62	126.20 ± 11.19	0.919	0.363
Increasing (%)	95.98 ± 22.54	86.80 ± 30.16	0.828	0.412

* *P* < 0.05, statistical significance.

## Discussion

LSS patients usually require bilateral decompression if they suffer from bilateral lower extremity symptoms. Total laminectomy decompression and intervertebral fusion with fixation (e.g., PLIF and TLIF) are conventional surgical techniques for the treatment of LSS, and this surgery can improve lower extremity symptoms through bilateral laminotomy for decompression. However, these techniques damage the bony structure and the attached ligaments behind the vertebral body, thus increasing the risks of secondary instability of the lumbar spine, lumbar spondylolisthesis, adjacent segment degeneration, and lumbar myofascial pain syndrome (2, 3). Along with the developments in spinal endoscopy and relevant surgical equipment, BEBD has been increasingly performed for LSS and achieves favorable clinical outcomes (4–9). Compared with conventional techniques such as PLIF and TLIF, BEBD causes less damage to the structures behind the spine and offers maximal protection for spinal stability. Besides, BEBD prevents a variety of complications that are otherwise common with open surgeries, and delivers several benefits, including reduced operation time, smaller incision, and faster recovery (10).

Many spinal endoscopic techniques, including BEBD, micro endoscopy and percutaneous endoscopic lumbar discectomy (PELD), have been reported for the treatment of LSS. All these approaches are considered to yield favorable clinical outcomes (6–8, 12–14). Eun et al. (12) reported a similar decompression effect using BEBD vs. micro endoscopy, although the former involved a smaller incision. Pranata et al. (13) reported similar clinical outcomes achieved by BEBD and micro endoscopy. However, BEBD showed greater benefits in terms of operation time, early ambulation, and the use of analgesics and offered clearer surgical view. Compared with PELD, Heo et al. (6) believed that BEBD achieved more thorough dural decompression, a smaller angle of MSF, and higher stability of the preserved FJ. Hwa et al. (14) believed that BEBD was similar to the conventional open surgeries from the anatomical perspective. BEBD demonstrates more advantages than PELD and the former better exposed the contralateral ligamentum flavum, intervertebral foramen, and nerve root. we emphasize that the optimal approach should be pathology-specific, with uniportal techniques maintaining advantages in far-lateral or extraforaminal cases. In addition, we emphasize that the optimal approach should be pathology-specific, with uniportal techniques maintaining advantages in far-lateral or extraforaminal cases. BEBD may be the first optional approach for minimally invasive surgery for LSS.

Dural tear is a complication in BEBD surgery, and its possible causes are related to the following factors. (1) Biomechanical constraints of endoscopic instruments - the limited working space and rigid instrumentation create challenging force vectors during manipulation near the dura; (2) Anatomical variations - including adherent epidural fat, thin or adherent dura mater (particularly in revision cases), and complex neural arch morphology that obscures visualization; (3) Learning curve dynamics - emphasizing the technical challenges in developing bimanual instrument control and depth perception through a narrow working channel. The analysis specifically highlights how our BEBD technique's standardized approach (including the 0° endoscope selection) helps mitigate these risks through optimized visual control and instrument ergonomics, while acknowledging that the complication remains an inherent risk.

BEBD has free of restrictions from ports, as both the observation and operation ports allow for higher flexibility and larger manipulation space. The surgical view thus offered is broader, and the positions and orientations of the ports can be adjusted as appropriate for contralateral undermining decompression to achieve better clinical outcomes (4, 12, 15–18). Among our cases, LRDR were sufficiently decompressed on both the ipsilateral and contralateral sides. The nerve root compression was relieved, and DSCAER increased significantly than before (95.19%). The dura mater and bilateral nerve roots were fully exposed after bilateral decompression in all 51 patients in our study. The symptoms were much improved after surgery, accompanied by a significant reduction in the VAS scores for the lower back and leg pain and ODI than before (*P* < 0.05). Besides, such a decrease persisted over time (*P* < 0.05). During the last follow-up, the patients achieving an excellent and good outcome rate of 88.24% according to the modified MacNab criteria.

By a unilateral approach, BEBD causes less damage to soft tissues and paravertebral muscles (19). Furthermore, BEBD allows for a smaller amount of FJ resected and therefore preserves enough bony structure to maintain lumbar spine stability, which, in turn, is conducive to early ambulation and faster functional recovery of the lumbar spine (20). Heo et al. (6) believed that when MFSA was smaller than 90°, its impact on the FJ stability was the minimum. In the present study, the average MFSA on the ipsilateral side was 86.58°, which was below 90°. The nerve roots in the lumbar spine are mostly located inferiorly or medially to the medial border of the superior FJ. Intraoperative exploration of the medial border of the superior FJ plus undermining decompression was performed in this study to offer better protection for FJ. If sufficient relaxation of the nerve root was confirmed, discectomy may be avoided, if possible, to ensure the stability of the structure in front of the lumbar spine. This is effective to prevent iatrogenic injury of intervertebral disc or accelerated degeneration (21). The postoperative 3D CT of the lumbar spine showed that the average residual rate of FJ on the ipsilateral side was above 70%, with only a small amount of FJ resected on the contralateral side (below 7%). Postoperative dynamic x-ray imaging of the lumbar spine showed that the average motion range of the operated segment was 6.5°, and there was no significant difference compared with the preoperative finding (*P* > 0.05). It can be seen that BEBD had no apparent impact on lumbar spine stability.

This study has certain limitations, primarily the relatively short follow-up duration, which may introduce potential bias in assessing the long-term outcomes of both surgical approaches. This discrepancy in observational periods introduces potential bias when evaluating durability and late complications, as longer-term outcomes for BEBD. Future investigations should incorporate larger patient cohorts and extended follow-up periods to validate the durability and comparative efficacy of these techniques over time.

## Conclusions

BEBD demonstrated significant clinical improvements and favorable radiological findings in patients with LSS, characterized by a marked enhancement of DSCAER postoperatively. The technique effectively preserved FJ integrity, contributing to maintained lumbar spinal biomechanical stability. Additionally, BEBD achieves effective bilateral decompression while maintaining lumbar biomechanical integrity, supporting its role as a reliable minimally invasive option for LSS. Notably, the absence of significant correlations between radiographic parameters and clinical outcomes suggests multifactorial determinants of postoperative recovery.

## Data Availability

The original contributions presented in the study are included in the article/Supplementary Material, further inquiries can be directed to the corresponding author.
